# Local Synthesis of Carbon Nanotubes in Silicon Microsystems: The Effect of Temperature Distribution on Growth Structure

**DOI:** 10.3390/ma6083160

**Published:** 2013-07-26

**Authors:** Bao Q. Ta, Tormod B. Haugen, Nils Hoivik, Einar Halvorsen, Knut E. Aasmundtveit

**Affiliations:** Department of Micro and Nano Systems Technology (IMST), Vestfold University College (HiVe), P.O. Box 2243, Tonsberg N-3130, Norway; E-Mails: quoc.bao@hive.no (B.Q.T.); tormod.haugen@student.hive.no (T.B.H.); nils.hoivik@hive.no (N.H.); einar.halvorsen@hive.no (E.H.)

**Keywords:** carbon nanotubes, defects, disorder, local synthesis, scanning transmission electron microscopy

## Abstract

Local synthesis and direct integration of carbon nanotubes (CNTs) into microsystems is a promising method for producing CNT-based devices in a single step, low-cost, and wafer-level, CMOS/MEMS-compatible process. In this report, the structure of the locally grown CNTs are studied by transmission imaging in scanning electron microscopy—S(T)EM. The characterization is performed directly on the microsystem, without any post-synthesis processing required. The results show an effect of temperature on the structure of CNTs: high temperature favors thin and regular structures, whereas low temperature favors “bamboo-like" structures.

## 1. Introduction

**Figure 1:**
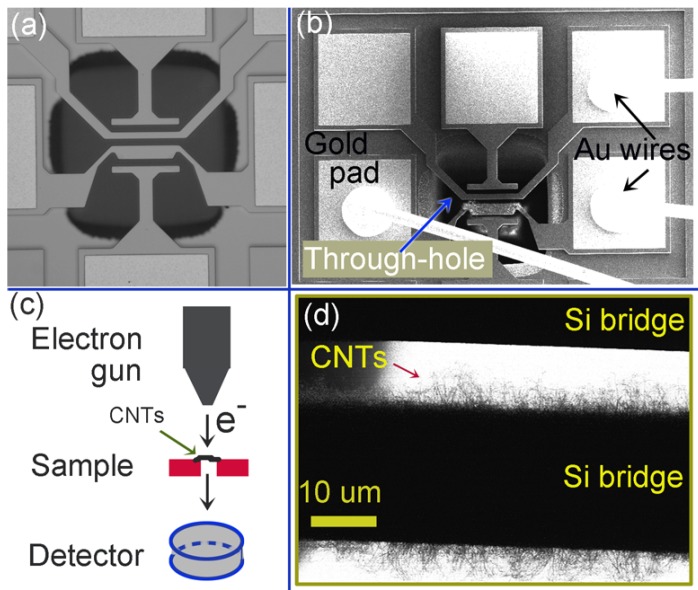
(**a**) An optical micrograph of a microsystem for locally synthesizing and direct integrating of carbon nanotubes (CNTs); (**b**) An SEM micrograph of the microsystem; (**c**) An illustration of the scanning electron microscopy [S(T)EM] characterization of locally grown CNTs; and (**d**) An S(T)EM image of the region for CNT growth in (**b**).

Carbon nanotubes (CNTs) have been a subject of intense studies since their discovery more than two decades ago. They have shown great potential in various fields of electronic, optical, mechanical, chemical and thermal applications. The integration of CNTs into MEMS and CMOS enables novel devices, such as CNT-based Field Effect Transistors, CNT-based memory and CNT-based sensors [1,2,3]. The synthesis of CNTs requires a high temperature, which can damage or change the properties of components in the microsystem or CMOS circuit. An alternative is to produce CNTs in a separate process and, then, individually place CNTs at specific locations in the microsystem. This method, however, has poor scalability and a high cost. The local synthesis and direct integration method [4,5,6,7,8] has a superior advantage that it localizes the hot region for CNT growth, and maintains the surroundings at room temperature. In addition, this method allows the control of the CNT orientation by using an electric field. More importantly, the resulting Si/CNTs/Si system can work as a gas sensor without any further processing for individual dies. Previous work demonstrated this synthesis technique [5] and a sensitivity of the resulting Si/CNTs/Si system to ammonia NH_3_ [6], showing the potential of a single-step, low-cost, wafer-level, CMOS/MEMS-compatible process for fabricating functional CNT-based devices. It is known that the physical/chemical properties of CNTs are closely related to the structures of the tubes. For example, a CNT can be metallic or semiconducting depending on the chirality. The defects in CNTs can alter the chemical reactivity, mechanical strength, absorption characteristics, and electronic transport of the CNTs. Therefore, the characterization of the CNT structure and defects are not only essential for the optimization of the synthesis process, but also for the applications of the final system. For a Si/CNTs/Si system fabricated by the local synthesis and direct integration method, the characterization of CNTs should neither take the CNTs out of the system nor cause any change or damage. In the current study, a customized design of a microsystem with a through-hole in the Si below the growth location of CNTs has enabled the transmission mode imaging by using a S(T)EM (Hitachi S-5500, Tokyo, Japan), as depicted in Figure 1. S(T)EM operates at low acceleration voltage, thus inducing less changes in the CNTs, compared to TEM [9,10]. In addition, the S(T)EM imaging of CNTs is performed directly without need for complicated steps of the sample preparation, and thus avoiding damages in CNTs. This report focuses on the characterization of the structure and defect of the locally grown CNTs, and on their relationship with the growth temperature.

## 2. Experimental Section

### 2.1. The Synthesis Process

**Figure 2:**
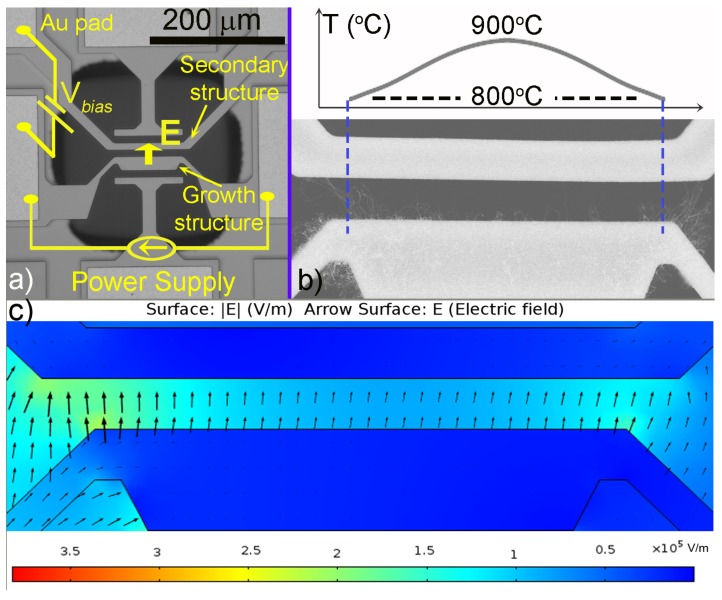
(**a**) Schematic illustration of the local synthesis and direct integration of CNTs into microsystems; (**b**) Simulated temperature profile on the growth microstructure; and (**c**) The local electric field between the two microstructures prior to the synthesis of CNTs (Finite element (FEM) simulation).

The CNTs were locally grown on a suspended Silicon-on-Insulator (SOI) microstructure at room temperature in a closed chamber with a pressure of 300 Torr. The principle of the synthesis method is described elsewhere [5]. Briefly described, the synthesis structure consists of two suspended microstructures (named growth microstructure and secondary microstructure). The growth microstructure was locally heated by joule heating to a desired temperature (900 °C) to initiate the nanotube growth. The carbon feedstock was a mixture of acetylene (C_2_H_2_) and a carrier gas (argon), both with a flow rate of 50 ccm. A 3-nm-thick layer of iron (Fe) was deposited by thermal evaporation on top of the growth microstructure prior to the synthesis. When the growth microstructure is heated, this layer will be transformed into nanoparticles to catalyze the CNT growth. A constant DC bias voltage was applied between the two microstructures to establish the electric field to orient the CNTs from the growth microstructure to the secondary microstructure. The circuitry is sketched in Figure 2a. Figure 2b shows the temperature gradient on the growth microstructure, due to joule heating. Figure 2c presents a simulation of the local electric field prior to the CNT growth; the field is quite homogeneous within the gap between the two microstructures, but not at the corners.

During the synthesis process, by measuring the electric current between the two microstructures, any CNT connection was detected as a jump in the measured current. The first CNT connection was normally established about 15–30 s after C_2_H_2_ was introduced. The CNT growth rate was, thus, about 1.5–3 µm/s, since the distance between the two microstructures is 10 µm.

### 2.2. The Characterization Process

Ten samples were characterized. All images were taken using a Hitachi S-5500 (a high resolution in-lens field-emission SEM, equipped with transmission detectors). The S-5500 was operating at 30 kV, and a bright-field (BF) detector was used.

## 3. Results

**Figure 3:**
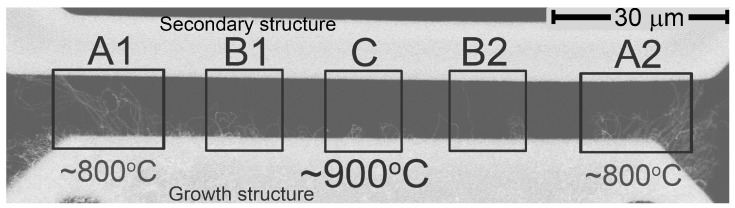
An SEM micrograph of the locally grown CNTs in the gap between the two microstructures. The marked regions were selected for further investigations, because the growth temperature varies. The density of CNTs is lowest at the highest-temperature Region (**C**), and highest at the lowest-temperature Region (**A**). Temperature in each region is estimated from simulation and one-time experimental calibration.

Figure 3 shows an SEM micrograph of a resulting Si/CNTs/Si system. The marked regions were chosen for further investigations because the growth temperature varies. Region C is at the center of the growth microstructure, where the temperature is highest (∼900 °C). Regions A (1 and 2) are at the corners of the growth microstructure, where the temperature is lowest (∼800 °C, shown in Figure 2). Region B (1 and 2) is in between A and C, where the temperature is intermediate (∼850 °C).

We observed that the density of CNTs was lowest at the highest-temperature Region (C) and highest at the lowest-temperature Region (A). The proportion of CNTs connecting the two microstructures, however, was found to be highest at Region C, and lowest at Region A.

**Figure 4:**
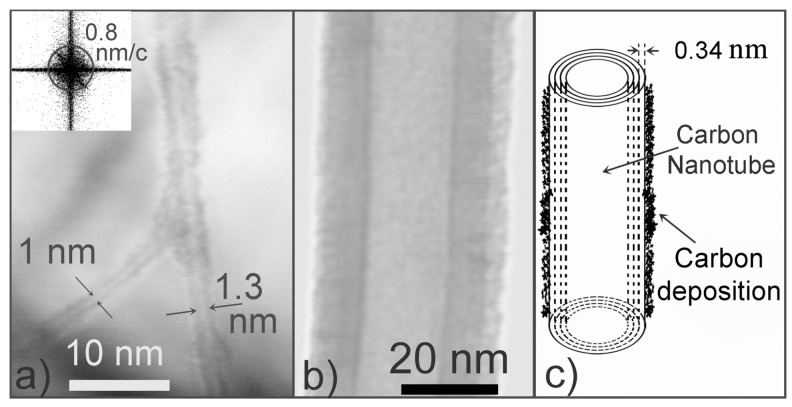
(**a**) and (**b**) S(T)EM images of CNTs at the highest-temperature Region (C, ∼900 °C); inset: FFT power spectrum of (**a**), indicating that the resolution of the image is ∼0.8 nm; and (**c**) Illustration of a CNT with carbon contamination deposited on the sidewall.

Figure 4 presents several S(T)EM images of CNTs at the highest-temperature Region (C). Thin CNTs with diameters of ∼1 nm were observed (Figure 4a). The image was captured at the highest magnification (×2000 k) of the S-5500, and the image resolution was ∼0.8 nm (indicated by the FFT (Fast Fourier Transform) power spectrum). As this resolution is more than twice the distance between neighboring tubes in a multi-walled CNT (∼0.34 nm [11]), we are not able to distinguish a single-walled CNT from a multi-walled CNT. Neither can we count the number of constituting tubes of a multi-walled CNT. Figure 4b presents a large-diameter CNT that appears to have three parts with different contrasts. The difference in contrast is due to the difference in the concentration of carbon atoms along the line of the transmitted electron beam (partly absorbed). The innermost bright part is interpreted as the hollow core, whereas the dark part is the overall wall thickness of a multi-walled CNT because the electron beam is less absorbed through the hollow core than through the wall of the CNT. The apparent inner and outer diameters of the CNT are ∼17 nm and ∼33 nm, respectively; and the apparent wall thickness is ∼8 nm. We interpret the outermost bright part as the carbonaceous contamination, which is a common product in thermal CVD growth. The carbonaceous deposit on the CNT sidewall is apparently non-uniform: ∼4 nm thick on the right side, ∼1–2 nm thick on the left side.

**Figure 5:**
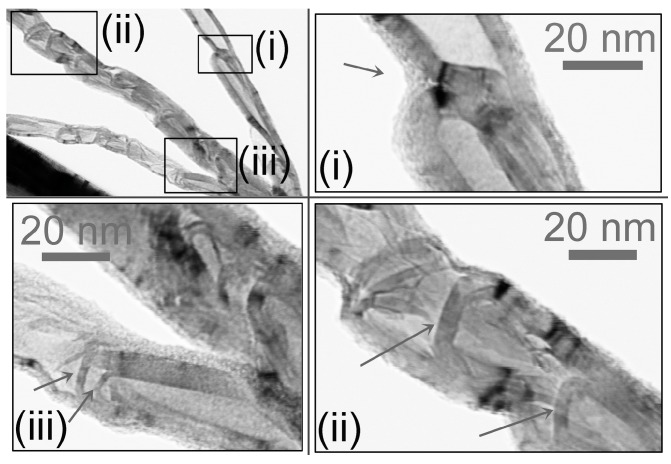
“Bamboo-like" CNTs are mainly produced at the lowest-temperature Region (A1 & A2, ∼800 °C). The “bamboo-like" CNT consists of hollow, capped compartments along the length. Two types of compartments were observed: “off-axis" (*i.e.*, the axis of the compartment is non-parallel to the main axis of the CNT) and “on-axis" (*i.e.*, the axis of the compartment is parallel to the main axis of the CNT). CNT-(i) is “off-axis"; CNT-(ii and iii) are “on-axis". The arrows indicate the compartments.

Figure 5 shows typical structures of CNTs grown at the lowest temperature Region (A). The CNTs appear to have a “bamboo-like" structure, which consists of hollow, closed compartments along the length of the CNTs. The bamboo compartments of CNT-(i) are apparently “off-axis" (*i.e.*, the axis of each compartment is non-parallel to the main axis of the CNT) . The compartments of the CNT-(ii) and CNT-(iii) seem to be “on-axis " (*i.e.*, the axis of each compartment is parallel to the main axis of the CNT). At Region A of all the samples, more than 50% of CNTs had a “bamboo-like" structure.

The structure of the compartments varies. In CNT-(i), each compartment has a hemispherical cap, and the wall thickness is uniform from the cap to the body. In CNT-(ii), each compartment has a flat cap traversing the central tube, and the inner diameter decreases from the cap to the body. In CNT-(iii), each compartment has a conical, flat-topped cap.

**Figure 6:**
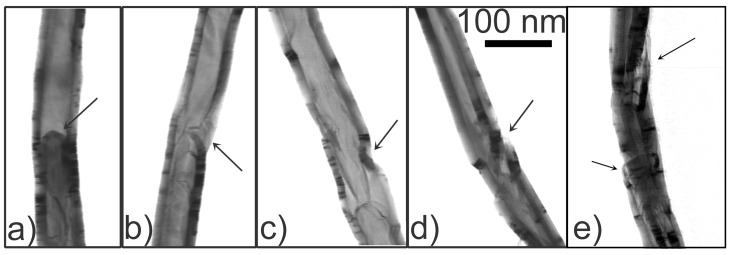
S(T)EM images of a CNT at the intermediate-temperature Region (B). The CNT has a fairly uniform diameter, but has several broken sites along the tube length. From left to right are the images presenting different segments along the CNT, in the order from the growth microstructure to the secondary microstructure. (**a**) At the segment closest to the growth microstructure: a compartment with a hemispherical cap inside the CNT was observed, but this is not a common case; (**b**)–(**d**) At further segments of the CNT: there are several broken and bent sites. Broken sites cause the CNT to bend towards one side. The arrows indicate the broken sites; and (**e**) At the furthest segment from the growth microstructure: the broken sites are larger and more defects are present.

Figure 6 presents several S(T)EM images of a CNT at the intermediate-temperature Region (B). The CNT has a fairly uniform diameter, but has several broken and bent sites along the length. From left to right are the images presenting different segments along the same CNT, in the order from the growth microstructure to the secondary microstructure. In the segment closest to the growth microstructure, a compartment with a hemispherical cap inside the CNT was observed (Figure 6a), but this is not a common case . In further segments of the CNT, there were several broken sites that cause the CNT to bend to one side (Figure 6b–d) , and this is a common case. In the furthest segment (Figure 6e), the broken sites are larger and more defects are present.

## 4. Discussion

Our result shows that the density of CNTs is lower at the Region with a higher temperature. This result is correlated to the growth density of CNTs on the surface of the growth microstructure, which is investigated statistically in another paper from our group appearing in this issue [12]. In our experiment, the Fe nanoparticles were created from the Fe film when locally heating the growth microstructure. According to Ramappa *et al.* [13] , the diffusion of Fe into SiO_2_ and Si occurs at temperatures ranging from 700 to 1100 °C; and the diffusivity follows the Arrhenius relationship. At a higher temperature, the rate of Fe diffusion in SiO_2_ and Si is higher, thus providing lower density of Fe nanoparticles. A lower density of Fe nanoparticles, in turn, results in a lower density of CNTs.

The density of CNTs at Region C is lowest; however, the proportion of CNTs connecting the two microstructures is highest. Since the growth temperature at Region C is highest, the grown CNTs are thinnest. Our statistical study, which has been submitted to the Applied Physics Letter [14] , shows that CNTs with smaller diameters are more straight and well-aligned to the electric field. The highest degree of straightness and alignment of CNTs at region C, in turn, gives the highest proportion of CNT connections.

We observed no trend in the length of grown CNTs *versus* the growth temperature. The growth rate of CNTs in CVD synthesis probably increases with the temperature [15] ; however, in our experiments, there were some other factors determining the CNT length. For example, a CNT may stop growing when it makes contact with the secondary microstructure. Large-size defects on the CNT body may cause the CNT to be broken or bent, thus terminating the CNT growth or reducing the growth rate.

Each Regions (A–C) appears to have distinct CNT structures. At the highest-temperature Region (C), the growth of thin and regular CNTs dominates. At the lowest-temperature Region (A), the “bamboo-like" CNTs dominate (>50% of the total number of CNTs in this Region). At the intermediate-temperature Region (B), CNTs have a fairly uniform diameter along the CNT length, but have several broken and bent sites. We note that the growth temperature is the dominant factor for the above distinction in the CNT structure, because other synthesis conditions are the same (and the local electric field has not been found to affect the structure of CNTs). Our experiments show that the degree of order in the CNT structure increases as the temperature increases. Sengrupta *et al.* [16] and Lee *et al.* [17] have found similar results. Our hypothesis is that a higher temperature provides a higher mobility of the carbon atoms and, thus, favors a reorganization process that increases the degree of order during CNT growth .

The structure of the contamination on the CNT, shown in Figure 4, is not yet identified. Harris *et al.* [18] have shown similar transmission images of CNTs and, by cross-sectional TEM, revealed that the contamination is mainly amorphous carbon. Amorphous carbons and mesoscopic graphitic carbons are known as common products in thermal CVD growth [19]. Beyond affecting the appearance of CNTs in the electron microscopy, the contamination can substantially change the chemical and physical properties of CNTs. For instance, amorphous carbon has higher reactivity, and a higher variety of functional groups than pristine CNTs; they have different gas absorption properties. Cantalini *et al.* [20] reported that the presence of amorphous carbon improved the electric response of the CNTs to NO_2_ gas.

The presence of “bamboo-like" CNTs suggests that the continued growth and nucleation of CNTs occurred [21]. Growth models for “bamboo-like" structures have been previously proposed by Saito *et al.* [22] and Kovalevski *et al.* [23] . Saito *et al.* [22] suggested that during growth phase of a tip-growth CNT, the accumulated stress at the interface between the CNT and the catalyst particle would “push" the CNT or catalyst particle away from each other, leaving a fresh surface of catalyst for another nucleation process. Kovalevski *et al.* [23] proposed that when the catalyst particle moves away from the existing CNT at a lower rate than the growth of new graphitic layers, “bamboo-like" structures will be formed. In the former model, the cap or tip of the bamboo compartment would orient in the same direction as the growth direction of the CNT, *i.e.*, the direction towards the secondary microstructure. In the latter model, the cap or tip of the bamboo compartment would orient in the opposite direction, *i.e.*, the direction towards the growth microstructure. Since the CNT growth rate in our study is very high (∼1.5–3 µm/s, which is ≳2 orders higher than other CVD methods [24,25,26,27,28,29]), the relaxation may not occur, and high stresses are likely to be created. In addition, the bamboo compartments in our CNTs oriented towards the secondary microstructure. Therefore, the growth of our “bamboo-like" CNTs might follow the former model (Saito *et al.* [22]). This, in turn, suggests a way to obtain more regular structures: reduce the stresses during CNT growth. A possible solution is to reduce the CNT growth rate by reducing the chamber pressure. Li *et al.* [30] claimed that at low pressure the CNTs have completely hollow cores, whereas at high pressure (>200 Torr), the “bamboo-like" CNTs are produced, and the density of bamboo compartments increases as the pressure increases .

Regarding the formation of broken sites in CNTs ( shown in Figure 6), we have two hypotheses. The first hypothesis suggests that the CNT growth follows the following sequence: tip-growth of the CNT initiates, then empty sites occur (probably due to uneven deposition of carbon), and finally, the CNT re-forms the wall and continues to grow. The second hypothesis suggests that broken sites originate from point defects in the body of the CNT, but later develop into larger sites due to high stresses during the growth. In our work, as there are high stresses in the CNTs during the growth (as discussed in the previous paragraph), the second hypothesis is more likely to be true .

Generally, defects and disorder are produced in almost all synthesis methods. In rapid and non-equilibrium growth process, such as the local synthesis in the current study, the degree of order in the CNT structure is rather low. Moreover, the local temperature gradient along a CNT during the growth can create different defects along the same CNT, as observed in Figure 6. Redcay *et al.* [31] have observed a similar effect in the growth of germanium nanowire.

The defects and disorder in CNTs usually decreases the controllability and repeatability of the synthesis process, but are beneficial for some applications. An example is the importance of defects and disorder in the chemoresistance of CNTs [19]. Reactive defects help enhance the charge transfer and the chemisorption between a CNT and gas molecules, thus improving the response of CNT-based gas sensors [32,33]. Therefore, future studies may focus on the sensor applications of the grown CNTs, rather than trying to minimize the degree of defects and disorder.

## 5. Conclusions

The structure and defects of the locally grown CNTs have been investigated along the heated microstructure. A correlation between the growth temperature and the structure of CNTs is revealed: the degree of order in the CNT structure increases as the growth temperature increases. The local synthesis method used in our study ensures the identical nature of the synthesis conditions, except for the local temperature at different Regions in the heated microstructure. The most regular CNTs grow at the highest-temperature Region. The “bamboo-like" CNTs dominate at the lowest-temperature Region. At the intermediate-temperature Region, CNTs have a fairly uniform diameter, but have several broken and bent sites along the nanotube length .
